# Messenger App–Based Information Provision for Promoting Social Participation to Enhance Well-Being Among Community-Dwelling Adults: Randomized Controlled Trial

**DOI:** 10.2196/57205

**Published:** 2024-11-29

**Authors:** Atsushi Nakagomi, Noriyuki Abe, Yu-Ru Chen, Kazushige Ide, Shuhei Kobayashi, Masamichi Hanazato, Katsunori Kondo

**Affiliations:** 1 Center for Preventive Medical Sciences Chiba University Chiba Japan

**Keywords:** messenger app, event information, happiness, social participation, messenger, app, well-being, adults, aging, randomized controlled trial, RCT, information technology, social activity, Japan, urban community, health information, control group, multivariable regression, life satisfaction, digital intervention, community-dwelling

## Abstract

**Background:**

Engaging in social activities, interacting with peers, and participating in community events may promote health and well-being. Recently, interventions leveraging information and communications technology have emerged as potent tools for promoting social connections and well-being. Particularly, messenger apps have become an integral part of our daily lives, facilitating communication, information dissemination, and social interaction. However, there remains a gap in the literature regarding the utilization of widely adopted messenger apps for this purpose.

**Objective:**

This study aimed to evaluate the impact of messenger app–based information provision aimed at promoting social participation on the enhancement of subjective well-being among Japanese community-dwelling adults.

**Methods:**

A 2-arm, parallel-group randomized controlled trial was conducted from October 2022 to January 2023 in the Kashiwa-no-ha campus area, Japan—an urban community with active local events. A total of 358 community-dwelling adults who use messenger apps daily were recruited for the study. Of these, 235 (65.6%) participants completed the follow-up survey. Participants were randomly assigned to either the intervention group, receiving the health benefits of social participation and information about local events or spots via a messenger app, or the control group, receiving general health information. The primary outcome was subjective happiness after the intervention, measured on an 11-point scale ranging from 0 (Unhappy) to 10 (Happy). Secondary outcomes included life satisfaction, meaning of life, purpose in life, and participation in local events. The outcomes were analyzed with *t* tests (2-tailed) and multivariable regression based on the intention-to-treat method.

**Results:**

After the intervention, the intervention group reported a mean happiness score of 7.7 (SD 1.7), while the control group reported a score of 7.5 (SD 2.0), with no statistically significant difference (*P*=.40). Multivariable linear regression analysis adjusted for baseline outcome values and covariates showed that the coefficient of the intervention for life satisfaction was 0.30 (95% CI –0.07 to 0.68; *P*=.12), while that for meaning of life was 0.33 (95% CI –0.03 to 0.70; *P*=.07). There was no significant difference in event participation rates between the 2 groups during the study period (*P*=.22). However, 82.2% (102/124) of the intervention group acknowledged the utility of the event information provided.

**Conclusions:**

Messenger app–based information provision did not yield a significant increase in subjective happiness, while there was a positive but not significant trend in life satisfaction. The findings underscore the need for more intensive intervention in future studies to harness the potential of digital interventions.

**Trial Registration:**

UMIN Clinical Trials Registry UMIN000049047; https://tinyurl.com/2zzrrae8

## Introduction

Happiness, an aspect of subjective well-being, has become a major topic in public health over the past decades [1]. It includes not only momentary joy but also the overall sense of positive effect that individuals experience in their lives. Recent research has underscored the influence of well-being, including happiness, on both mental and physical health outcomes [2,3], making it a public health concern. An example of this is the World Happiness Report—an annual publication by the Sustainable Development Solutions Network that ranks countries based on their citizens’ self-reported well-being [4]. This report not only emphasizes the importance of happiness as a global metric but also encourages nations to prioritize well-being in their policies and agendas. The prominence of such a report on the global stage underscores the growing recognition of happiness as a crucial component in assessing a nation’s health and overall success.

Engaging in social activities, interacting with peers, and participating in community events may promote multidimensional health and well-being [5-8], including subjective happiness [9,10]. Thus, the promotion of social connections can be a strategy to enhance the subjective happiness of populations.

Recently, interventions leveraging information and communications technology have emerged as potent tools to promote social connections [11-13]. Mobile apps, in particular, are widely accessible and commonly tool, making them a potential candidate for information and communications technology–based interventions. For example, a mobile app designed and implemented in the municipality of Enschede, the Netherlands, aimed to promote social participation among older adults within the community [14]. The app provided older individuals with access to a home page comprising 6 distinct features: an inbox for messaging, news updates, activity listings, informative resources, helpful tips, and friend connections. Notably, the app demonstrated favorable usability among the older population and positively changed the mental component of their quality of life. However, it is important to acknowledge the limitations inherent in the aforementioned study, as it was observational rather than a randomized controlled trial (RCT), which may have affected the strength of the conclusions drawn.

Messenger apps also have potential in the abovementioned context [15,16]. They have become an integral part of our daily lives, facilitating communication, information dissemination, and social interaction. These platforms, with their widespread reach and user-friendly interfaces, present a unique opportunity for public health interventions. LINE (LY Corporation), for example, is a messaging app originally developed in Japan, offering a range of communication tools, including SMS text messaging, voice and video calls, and group chats. Beyond its primary function as a communication platform, LINE has expanded its services to include features tailored to businesses. One notable feature is the “LINE Business Account,” which allows businesses to disseminate information, promote products or services, and engage with users directly through the app. Given LINE’s immense popularity in Japan [17], with over 95 million users, numerous businesses use the platform for promotional activities, while local governments harness its reach for public service announcements, community engagement, and other civic initiatives.

Despite the potential of messenger app interventions in promoting social connections and, in turn, subjective happiness, there remains a gap in the literature regarding the utilization of widely adopted platforms, such as LINE, for happiness promotion in the Japanese context. Although LINE’s vast user base and multifunctionality make it a promising tool for public health initiatives, its potential in disseminating information regarding local events and fostering social connections to promote subjective happiness has not been extensively explored. The aim of this study was to investigate whether information provision via LINE, pertaining to local events and recommended spots emphasizing the importance of social connections, can promote subjective happiness. We conducted an RCT to examine whether leveraging a popular messaging platform can enhance subjective happiness among urban Japanese residents.

## Methods

### Trial Design

This trial was an RCT with parallel groups, conducted in the Kashiwa-no-ha campus area, Kashiwa City, Japan, from October 14, 2022, to January 31, 2023. Eligible participants were randomly assigned (allocation ratio 1:1) to the intervention group or control group. Results from the trial are reported in line with the CONSORT-EHEALTH guidelines [18] (Multimedia Appendix 1).

### Study Area

The Kashiwa-no-ha campus area is located within an hour of Tokyo. This area represents a blend of modern urban planning and community-centric development, frequently hosting diverse local events that attract residents and tourists alike. However, this area faces challenges in terms of information dissemination. Multiple sectors, from educational institutions to local governments, independently release event details, leading to fragmented communication. This decentralization complicates navigation for residents, highlighting the need for a unified platform to centralize event and community information.

### Sample Size

To determine the sample size, power analysis was conducted using GPower (version 3.1; Heinrich Heine University Düsseldorf), and the estimated effect size was based on the results of a similar study [19]. The required sample size, with an effect size of 0.40 on happiness and an α level of 0.05, was determined to be 200. Accounting for a 20% dropout rate, the plan was to achieve a final sample comprising 240 participants.

### Participants and Procedure

The LINE messaging app, launched in 2011, is of Japanese origin. Beyond SMS text messaging, it also offers voice calls, video calls, and official accounts. We created the official account “Shiawase (ie, happiness) City Kashiwa-no-ha” to navigate study participation and provide information to the intervention and control groups (Figure S1 in Multimedia Appendix 2).

Participants were eligible for the study if they lived or commuted to work in the Kashiwa-no-ha campus area and (1) were men and women aged over 18 years old, (2) were “LINE” mobile app users, and (3) had given consent for this study. Eligible participants were recruited via the posting of flyers seeking their participation in the project. We sent email invitations to various membership groups, in addition to holding study information sessions (both web based and in person) to explain the study protocols and procedures. The registration period was 1 month from the start of recruitment, and prospective participants were asked to register a “Shiawase (ie, happiness) City Kashiwa-no-ha” account for the study on the “LINE” mobile app through their smartphones. After registration of the account, participants were asked to tap a panel on their smartphone screen to access an external website, on which they read the explanations of the study, gave informed consent, and responded to a baseline survey on Google Forms.

Of the 376 eligible participants, 358 (95.2%) completed the baseline questionnaire on Google Forms, and 18 (4.8%) were excluded, mainly as a result of declining to participate. Following baseline evaluation, simple randomization was conducted, before, subsequently, the participants received information according to their assigned group for the 2-month period. After the intervention period, the participants were invited to respond to a follow-up survey on Google Forms.

### Randomization and Blinding

Computer-generated randomization was conducted on the LINE messaging platform after participants completed the baseline assessment. Participants were then automatically allocated to chat areas for the intervention and control groups, respectively (see Figure S1C in Multimedia Appendix 2 for the chat area for the intervention group). The study investigators were blinded to group allocation.

### Intervention Group

Participants who had been allocated to the intervention group received, for 2 months, information regarding local events (eg, hobby groups) and spots (eg, shops, parks) in the target area (Kashiwa-no-ha), as well as short articles describing the health benefits of social participation on LINE (Figure S1 in Multimedia Appendix 2). Participants also received event information once a week and short articles or recommended places to go 1-2 times a week. Event information was aggregated from the websites of commercial facilities and community websites in the Kashiwa-no-ha campus area and was updated every Monday. The events were categorized into (1) hobbies and volunteer activities, (2) health and sports activities, and (3) childcare and pets. The recommended spot information was based on the results of our pilot survey with Kashiwa-no-ha campus area residents, in which the residents provided their recommendations for indoor or outdoor spots (eg, parks and commercial facilities, etc). Short articles disseminated the findings on the importance of social participation in Japan [20,21]. To allow access to the details of this information, an external link was attached, navigating the participants to a web page that was available only to the intervention group. They could also visit the website by tapping a panel on their smartphone screens. The information was updated by NA.

### Control Group

Participants allocated to the control group received, for 2 months, general health information that did not include information about social participation. Participants received this information 2-3 times per week. The health information was presented in the form of headings from the Japanese Ministry of Health, Labour and Welfare’s website (e-health net [22]), with external links attached. The external links were on a web page that was accessible only to the control group. They could also visit the website by tapping a panel on their smartphone’s screen. The information was updated by NA.

### Measures

#### Primary Outcome

The primary outcome was subjective happiness (hedonic well-being), measured on an 11-point scale ranging from 0 (Unhappy) to 10 (Happy), during the follow-up assessment [1]. We used the following question: “In general, how happy do you usually feel?”

#### Secondary Outcomes

Our secondary outcomes were life satisfaction (evaluative well-being), meaning of life, and purpose in life (eudaimonic well-being) at the follow-up assessment [1]. We used the following questions or statements: “Overall, how satisfied are you with life as a whole these days? (0=Not satisfied at all, 10=Completely satisfied),” “Overall, to what extent do you feel the things you do in your life are worthwhile? (0=Not at all worthwhile, 10=Completely worthwhile),” and “I understand my purpose in life (0=Strongly disagree, 10=Strongly agree).” Changes in well-being measures between the baseline and follow-up assessments were also secondary outcomes.

In addition, we assessed the effectiveness of the intervention in promoting participation in local events. Participants were asked, as part of the follow-up assessment, about their attendance at events in Kashiwa-no-ha during the study period.

#### Covariates

We assessed age, gender, self-rated physical health, self-rated mental health, financial stability, and material stability as covariates in the additional analysis [23]. All variables were based on Google Forms questionnaires. We asked the following questions: “In general, how would you rate your physical health? (0=Poor, 10=Excellent),” “How would you rate your overall mental health? (0=Poor, 10=Excellent),” “How often do you worry about being able to meet normal monthly living expenses? (0=Worry all of the time, 10=Do not ever worry),” and “How often do you worry about safety, food, or housing? (0=Worry all of the time, 10=Do not ever worry).”

#### Feedback on Information Utility

After the intervention period, participants in the intervention group were asked to provide feedback on the utility of the information they received via LINE. Specifically, they were asked to choose the useful information, if any existed: local events, recommended spots, and the short articles about social connection and health.

### Incentives

The participants received an incentive of JP \1000 in e-money (approximately US $7) to complete both the baseline and follow-up questionnaires. Participants could choose to receive their e-money from Amazon, LINE Pay, PayPay Point, Google Pay, and so on.

### Statistical Analysis

Analyses were performed on an intention-to-treat basis among people who responded to the follow-up questionnaire. Means and SDs for the continuous variables and frequencies for the categorical variables were presented. We used a Student *t* test to compare the postintervention outcomes and changes in the outcomes from baseline to the postintervention time point between the control and intervention groups (significance level: *P*≤.05). In addition, we conducted multivariable linear regression in which the outcomes were postintervention values. In model 1, we adjusted for each baseline outcome (subjective happiness, life satisfaction, meaning of life, and purpose in life). In model 2, age, gender, self-rated physical health, self-rated mental health, financial stability, and material stability were added. All analyses were performed using STATA MP (version 18.0; Stata Corp).

### Ethical Considerations

Ethical approval for this study was obtained from the Ethics Committee at Chiba University (approval M10428). Informed consent was obtained from each participant. The protocol was registered in the UMIN Clinical Trials Registry with the identifier 000049047. Informed consent was obtained individually online before study participation, adhering to the principles of the Declaration of Helsinki. All data were deidentified to protect the privacy and confidentiality of participants. Participants received JP \1000 in e-money (approximately US $7) as compensation to complete the baseline and follow-up questionnaires.

## Results

**Figure 1:**
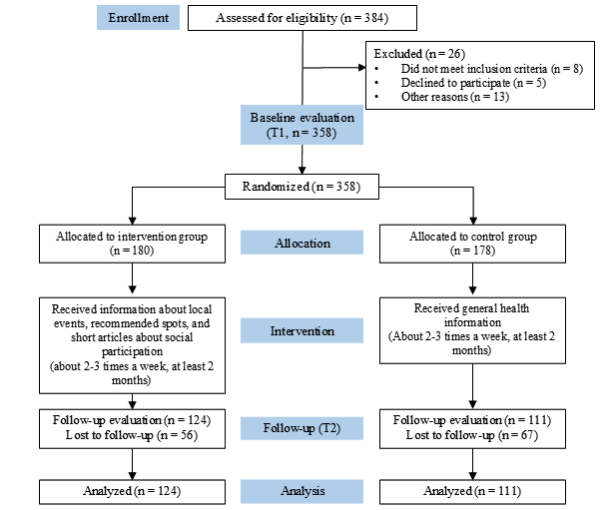
CONSORT (Consolidated Standards of Reporting Trials) flow diagram for study participants.

A total of 235 participants responded to the follow-up survey: 124 (52.8%) from the intervention group (56/180, 31.1% dropout rate) and 111 (47.2%) from the control group (67/178, 37.6% dropout rate; Figure 1). Table 1 summarizes the baseline characteristics of the participants. The mean age was 47.8 (SD 13.8) years, with 63.8% (150/235) of the sample comprising women. There were no significant differences in baseline characteristics and outcomes (all *P*>.05).

There was no difference in subjective happiness between the intervention group (mean 7.7, SD 1.7) and control group (mean 7.5, SD 2.0) after the 2-month intervention period (*P*=.40; Table 2). We also found no significant differences in life satisfaction, meaning of life, and purpose in life between the 2 groups (all *P*>.05).

Multivariable linear regression analysis, adjusted for baseline outcome values and covariates, showed that the coefficient of the intervention for life satisfaction was 0.30 (95% CI –0.07 to 0.68; *P*=.12) and that for meaning of life was 0.33 (95% CI –0.03 to 0.70; *P*=.07; Table S1 in the Multimedia Appendix 2). Our additional analysis, in which outcomes represented changes in well-being between baseline and follow-up, revealed similar trends (life satisfaction: intervention, mean 0.13, SD 1.54 vs control, mean –0.24, SD 1.77, *P*=.09; meaning of life: intervention, mean –0.20, SD 1.46 vs control, mean –0.51, SD 1.58, *P*=.12; Table S2 in the Multimedia Appendix 2).

There was no difference regarding participation in events in Kashiwa-no-ha during the study period between the intervention group (48/124, 38.7%) and the control group (52/111, 46.8%; *P*=.21). Most of the intervention group (102/124, 82.2%) found the event information useful, while only 11.3% (14/124) considered the short articles related to social connection and health useful (Table S3 in the Multimedia Appendix 2).

**Table 1:** Participants’ characteristics at baseline.

Characteristics	Total (N=235)	Intervention (n=124)	Control (n=111)	*P* value
Age (years), mean (SD)	47.8 (13.8)	47.8 (13.7)	47.9 (14.0)	.96
Gender (women), n (%)	150 (63.8)	79 (63.7)	71 (64.0)	.89
Physical health (0-10), mean (SD)	7.3 (1.8)	7.3 (1.8)	7.2 (1.8)	.62
Mental health (0-10), mean (SD)	7.3 (1.8)	7.2 (1.8)	7.3 (1.8)	.85
Financial stability (0-10), mean (SD)	6.2 (2)	6.1 (2.2)	6.3 (1.7)	.54
Material stability (0-10), mean (SD)	6 (2)	6.1 (2.2)	5.8 (1.8)	.33
Happiness (0-10), mean (SD)	7.8 (1.5)	7.9 (1.5)	7.7 (1.5)	.48
Life satisfaction (0-10), mean (SD)	7.6 (1.6)	7.5 (1.6)	7.6 (1.5)	.52
Meaning of life (0-10), mean (SD)	7.7 (1.7)	7.7 (1.8)	7.6 (1.7)	.81
Purpose in life (0-10), mean (SD)	7 (2.5)	6.9 (2.8)	7.1 (2.3)	.63

**Table 2:** Well-being at follow-up and Cohen d for between-group effect sizes.

	Intervention, mean (SD)	Control, mean (SD)	Cohen *d*	*P* value
Happiness	7.7 (1.7)	7.5 (2)	0.11	.40
Life satisfaction	7.6 (1.7)	7.4 (1.8)	0.14	.29
Meaning of life	7.5 (1.7)	7.1 (2)	0.20	.13
Purpose in life	6.4 (2.3)	6.3 (2.2)	0.03	.79

## Discussion

### Principal Findings

This study explored the efficacy of providing aimed at promoting social participation on the LINE messaging platform, emphasizing the importance of social connections for the promotion of happiness. Our findings suggest that the intervention did not significantly enhance subjective happiness and local events participation. While care should be taken in interpretation, there was a slight positive trend in life satisfaction and meaning of life among the intervention group compared with the control group, but this was not statistically significant.

The absence of a significant difference in subjective happiness between the intervention and control groups may be due to the lack of notable changes in event attendance between the 2 groups. This finding did not align with some studies showing the effectiveness of social participation apps [14,24]. Several factors could explain this finding. First, merely providing information might not have been sufficient to motivate individuals to participate in community events. Previous studies used some other factors, such as message boards and matching, which may promote communication and participation [14,24]. Second, the study did not comprehensively cover events and spots, which could have diluted its potential impact. Third, it is plausible that participants, given their proactive stance on improving their communities, as evidenced by their participation in this study, had already attended local events. The high local event participation rates (41.7% in the intervention group and 50% in the control group) further support this idea. The aforementioned pre-existing engagement may have attenuated the potential impact of the intervention.

Changing health behavior is a complex endeavor, often requiring deep-rooted shifts in mindset and habits. Many studies have harnessed theories, such as self-determination theory [25], to bolster motivation and drive behavioral change. However, promoting social participation presents unique challenges distinct from those seen with traditional health behaviors because the pro-health impacts of social participation are not common in the general population. Additionally, this study revealed that its participants did not highly value short articles linking social participation with health, thus indicating a potential disconnect in recognizing the health benefits of social engagement. Although effective strategies in health behavior change often employ features such as goal setting and feedback [26], these may not be directly translatable to the promotion of social participation. Specifically, the utility of goal setting and feedback diminishes if individuals do not first acknowledge and value the importance of social participation for their health. Additional motivational factors or behavioral nudges may be necessary to promote social engagement as a health behavior [27].

The observed trend of improvement in life satisfaction and the meaning of life, but not in happiness, among members of the intervention group offers an intriguing insight into the multifaceted nature of subjective well-being [1]. While happiness captures momentary emotional states influenced by daily experiences, life satisfaction and the meaning of life reflect more enduring aspects of well-being. Despite the intervention's emphasis on local events and the importance of social connections, it did not lead to an immediate increase in event attendance. The aforementioned finding suggests that the intervention may have influenced participants’ cognitive perceptions and evaluations without necessarily prompting an actual increase in social events attendance. This aligns with social contagion, demonstrating that attitudes, behaviors, and emotions spread in a network through the diffusion of information or the transmission of behavioral norms [28]. The consistent exposure to community opportunities, even if not directly acted upon, may have nurtured, within participants, a heightened sense of belonging or a feeling of being more integrated and informed about their community. This could, in turn, have contributed to a more favorable overall judgment of their lives, enhancing life satisfaction and the meaning of life, without altering momentary feelings of happiness. However, given these less robust findings, further research is needed to delve deeper into the differential impacts on various facets of subjective well-being and to understand the underlying mechanisms.

The fact that the majority of the intervention group members found the event information useful suggests the potential impact of tailored community information delivered through familiar platforms such as LINE in Japan. This positive reception indicates that the participants valued being informed about local events and opportunities, suggesting a latent desire for community engagement and connection. Leveraging popular communication tools to bridge the gap between residents and their local communities may drive more active participation in future initiatives and enhance well-being.

### Limitation

This study has several limitations that should be considered when interpreting the findings. First, the intervention’s duration (2 months) might have been too short to observe significant behavioral changes, such as increased event attendance. Second, the sample might have been skewed toward individuals who are already proactive regarding community engagement, given their residence in the area and willingness to participate in the study, thus potentially limiting the generalizability of the findings to a broader population. However, it is worth noting that those who did not participate in the study, potentially being less proactive, might have more room for improvement in terms of community engagement and well-being. The aforementioned suggests that future interventions could target this less-engaged demographic, as they might benefit more substantially from initiatives promoting community involvement and social connections. Third, the participants were relatively young and expected to be currently employed. Some of them might not have been able to attend the events. Fourth, we could not identify the actual event participation, merely relying on the responses to the questionnaire at follow-up assessment. Fifth, the coverage of the local information provided might not have been comprehensive, giving rise to the possibility of missing out on events or opportunities that could have been of interest to participants. It is essential to recognize that individuals are more likely to use and trust an information resource if they perceive it to be comprehensive and all-encompassing. Incomplete information might deter consistent engagement, emphasizing the need for future interventions to ensure thoroughness in information dissemination to maximize user trust and utilization. Sixth, we were unable to prevent control group participants from independently browsing information on the health benefits of social participation or local events. Seventh, we did not consider employment and membership with organizations in the analysis. Although this study is an RCT, these factors can affect the results and should be included in multivariable analysis. Finally, this study had a notable dropout rate. If participants in the intervention group who found the intervention beneficial were more likely to complete the follow-up survey, this could have led to an overestimation of the intervention’s effectiveness.

### Conclusions

In summary, while our study did not find that messenger app–based information provision impacted subjective happiness, the potential positive trend in life satisfaction and meaning of life warrants further exploration. Future research should consider longer intervention durations, the incorporation of behavioral change techniques, and comprehensive information on local events and spots to maximize the benefits of such digital interventions in promoting social participation and well-being.

## Appendix

Multimedia Appendix 1 CONSORT-eHEALTH checklist (V 1.6.1).

Multimedia Appendix 2 Supplementary data.

## Abbreviations

RCT: randomized controlled study

## Data Availability

The datasets analyzed during this study are available from the corresponding author on reasonable request.
