# Supplementary material for: Messenger App–Based Information Provision for Promoting Social Participation to Enhance Well-Being Among Community-Dwelling Adults: Randomized Controlled Trial
Source: J Med Internet Res. 2024 Nov 29;26:e57205. doi: 10.2196/57205 (PMC11645508; doi:10.2196/57205)
Supplement: Multimedia Appendix 2 [file jmir_v26i1e57205_app2.docx]

**Figure S1 The LINE official account “Shiawase (i.e. happiness) City Kashiwa-no-ha”**

1. The account’s top page


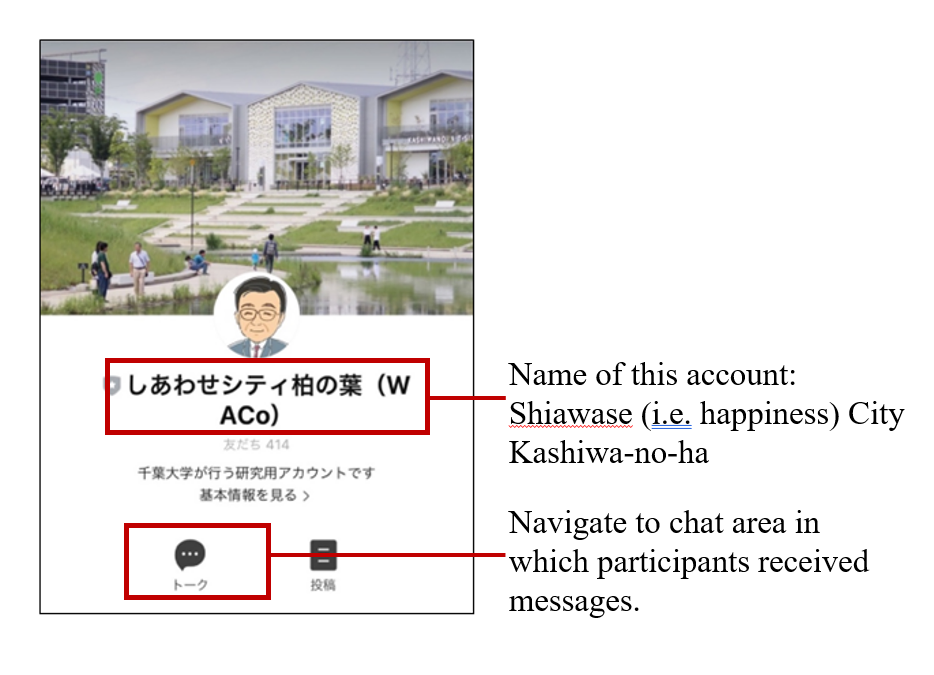


(b) Chat area before participating in this study


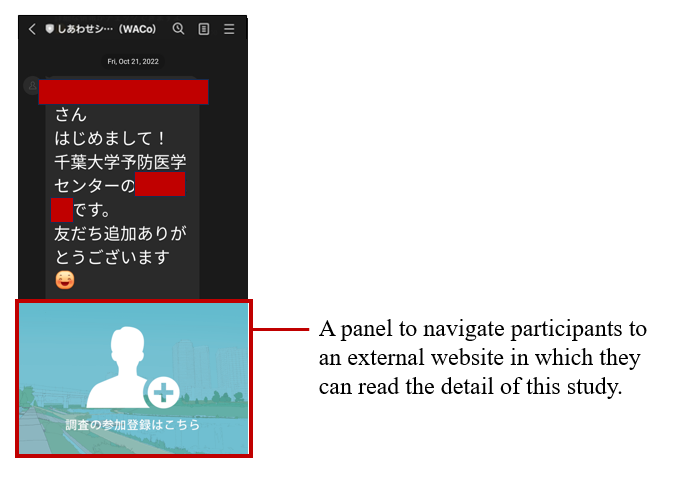


(C) Chat area during the study period


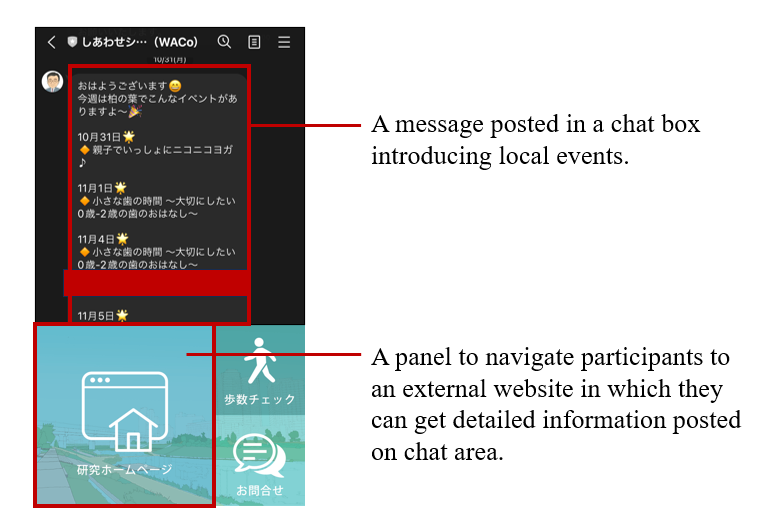


(d) The external websites for the intervention group.


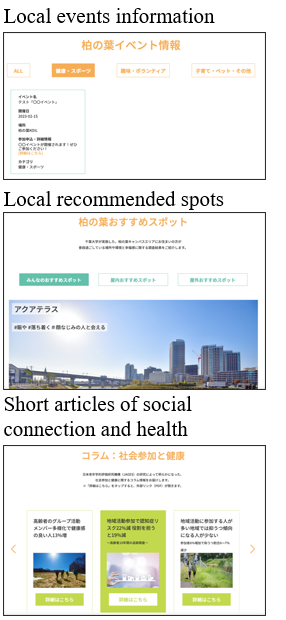


(e) The external websites for the control group.


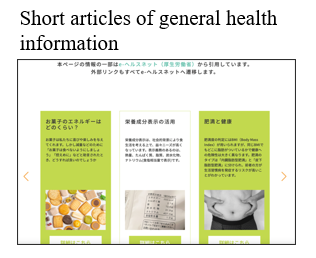


**Supplementary tables**

**Table S1.** Multivariable regerssion analysis in which outcomes were post-intervention values.

|  | Model 1 | |  | Model 2 | |
| --- | --- | --- | --- | --- | --- |
| Outcomes | Coefficient [95% CI] | *P* value |  | Coefficient [95% CI] | *P* value |
| Happiness | 0.09 [-0.27, 0.46] | .608 |  | 0.10 [-0.27, 0.46] | .595 |
| Life satisfaction | 0.31 [-0.07, 0.70] | .112 |  | 0.30 [-0.07, 0.68] | .115 |
| Meaning of life | 0.33 [-0.04, 0.69] | .077 |  | 0.33 [-0.03, 0.70] | .072 |
| Purpose in life | 0.11 [-0.46, 0.68] | .699 |  | 0.08 [-0.44, 0.60] | .759 |

CI: confidence interval

Model 1: adjusted for each outcome at baseline.

Model 2: adjusted for each outcome at baseline, age, gender, self-rated physical health, self-rated mental health, financial stability, and material stability.

**Table S2.** Changes in well-being and Cohen's d for between group effect sizes.

|  | Intervention, mean (SD) | Control, mean (SD) | Cohen's *d* | *P* value |
| --- | --- | --- | --- | --- |
| Happiness | -0.13 (1.41) | -0.19 (1.49) | 0.04 | .751 |
| Life satisfaction | 0.13 (1.54) | -0.24 (1.77) | 0.22 | .086 |
| Meaning of life | -0.20 (1.46) | -0.51 (1.58) | 0.21 | .117 |
| Purpose in life | -0.5 (3.22) | -0.74 (2.68) | 0.08 | .540 |

SD: standard deviation.

**Table S3.** Information that the participants in the intervention group (n = 124) considered useful.

|  |  | n (%) |
| --- | --- | --- |
| Event |  | 102 (82.3%) |
| Spots |  | 83 (66.9%) |
| Short articles of social connection and health |  | 14 (11.3%) |
